# Sertoli Cell *Wt1* Regulates Peritubular Myoid Cell and Fetal Leydig Cell Differentiation during Fetal Testis Development

**DOI:** 10.1371/journal.pone.0167920

**Published:** 2016-12-30

**Authors:** Qing Wen, Yuqian Wang, Jixin Tang, C. Yan Cheng, Yi-Xun Liu

**Affiliations:** 1 State Key Laboratory of Stem Cells and Reproductive Biology, Institute of Zoology, Chinese Academy of Sciences, Beijing, China; 2 University of Chinese Academy of Sciences, Beijing, China; 3 The Mary M. Wohlford Laboratory for Male Contraceptive Research, Center for Biomedical Research, Population Council, New York, New York, United States of America; Medical College of Wisconsin, UNITED STATES

## Abstract

Sertoli cells play a significant role in regulating fetal testis compartmentalization to generate testis cords and interstitium during development. The Sertoli cell *Wilms’ tumor 1* (*Wt1*) gene, which encodes ~24 zinc finger-containing transcription factors, is known to play a crucial role in fetal testis cord assembly and maintenance. However, whether *Wt1* regulates fetal testis compartmentalization by modulating the development of peritubular myoid cells (PMCs) and/or fetal Leydig cells (FLCs) remains unknown. Using a *Wt1*^-/flox^; *Amh*-Cre mouse model by deleting *Wt1* in Sertoli cells (*Wt1*^SC-cKO^) at embryonic day 14.5 (E14.5), *Wt1* was found to regulate PMC and FLC development. *Wt1* deletion in fetal testis Sertoli cells caused aberrant differentiation and proliferation of PMCs, FLCs and interstitial progenitor cells from embryo to newborn, leading to abnormal fetal testis interstitial development. Specifically, the expression of PMC marker genes *α-Sma*, *Myh11* and *Des*, and interstitial progenitor cell marker gene *Vcam1* were down-regulated, whereas FLC marker genes *StAR*, *Cyp11a1*, *Cyp17a1* and *Hsd3b1* were up-regulated, in neonatal *Wt1*^SC-cKO^ testes. The ratio of PMC:FLC were also reduced in *Wt1*^SC-cKO^ testes, concomitant with a down-regulation of Notch signaling molecules *Jag 1*, *Notch 2*, *Notch 3*, and *Hes1* in neonatal *Wt1*^SC-cKO^ testes, illustrating changes in the differentiation status of FLC from their interstitial progenitor cells during fetal testis development. In summary, *Wt1* regulates the development of FLC and interstitial progenitor cell lineages through Notch signaling, and it also plays a role in PMC development. Collectively, these effects confer fetal testis compartmentalization.

## Introduction

During embryogenesis, Sry (sex-determining region of the Y chromosome) expression in pre-Sertoli cells of XY individuals turns on a genetic cascade by directing the bipotential genital ridge to develop into the testis [1]. The onset of Sry expression leads to Sertoli cell aggregation, encircling germ cells to form testis cords which are then surrounded by peritubular myoid cells (PMCs) [for reviews, see [2–4]]. Between testis cords is the interstitium, inhabited by fetal Leydig cells (FLCs), uncharacterized interstitial progenitor cells, arterial and venous blood vasculature, lymphatic vessels, resident macrophages and nerve cells [for reviews, see [2–4]]. Thus, the differentiation, proliferation and movements of different testicular cell types are tightly coordinated to support fetal testis compartmentalization. Although the genetic networks and the testis cell types responsible for testis development are known [for reviews, see [2, 3, 5]], the cellular interactions that confer fetal testis compartmentalization remain unclear. Sertoli cell is thought to be the critical cell type that drives fetal testis compartmentalization [4], yet accumulating evidence has shown that FLCs and PMCs also play active roles in fetal testis development. Studies have shown that FLCs modulate Sertoli cell proliferation, and testis cord elongation and expansion via activin A [6]. PMCs also interact with Sertoli cells to deposit extracellular matrix components to form the basement membrane that defines the testis cords and interstitium [7]. However, whether Sertoli cells regulate PMC and FLC development to drive fetal testis compartmentalization is still unclear.

*Wt1* is a tumor suppressor and also an oncogene encoding at least 24 transcription factors involved in cell proliferation, differentiation, apoptosis and organ development [reviewed in [8, 9]]. Global knockout of *Wt1* in mice led to gonad agenesis and embryonic lethality [10]. In the testis, the Sertoli cell is the major cell type expressed *Wt1*, and Sertoli cell-specific deletion of *Wt1* using *Amh*-Cre was earlier reported to disrupt the formation of testis cords in fetal testis [11], likely mediated by a down-regulation of collagen α1(IV) and α2(IV) expression [12], and re-programming of Sertoli cells to Leydig-like cells [13]. However, it remained unknown whether Sertoli cell-specific deletion of *Wt1* would modulate differentiation and proliferation of FLCs and PMCs, which in turn perturbed testis compartmentalization during fetal testis development. In this study, we used *Wt1*^SC-cKO^ mice to perform a detailed analysis on the differentiation, proliferation and apoptotic status of PMCs, FLCs and their shared interstitial progenitor cells to unravel the role of *Wt1* in fetal testis development.

## Materials and Methods

### Mouse genetics

The use of mice for experiments reported herein was approved by the Animal Care Committee of the Institute of Zoology, Chinese Academy of Sciences. All mice were maintained in a C57BL/6;129/SvEv mixed background. *Wt1* ^+/flox^ mice [11] were mated with mice carrying the *Wt1*-null allele (*Wt1*^+/−^) [10] and *Amh*-Cre transgenic mice [14] to produce *Wt1*^-/flox^; *Amh*-Cre (*Wt1*^SC-cKO^) offspring. All mouse lines were provided by Dr. Fei Gao (Institute of Zoology, Chinese Academy of Sciences). DNA isolated from tail biopsies was used for genotyping by PCR to confirm Sertoli cell *Wt1* knockout (cKO) in fetal males as earlier described [10, 11, 14]. No difference was found among *Wt1* ^+/flox^, *Wt1* ^+/flox^; *Amh*-Cre, *Wt1*^-/flox^ or wild-type mice, thus the age-matched male mice (*Wt1* ^+/flox^, *Wt1* ^+/flox^; *Amh*-Cre or *Wt1*^-/flox^) served as the corresponding control mice of *Wt1* ^-/flox^; *Amh*-Cre.

### Immunofluorescence (IF) and immunohistochemistry (IHC) analysis

IF was performed using both paraffin and frozen sections, and IHC using paraffin sections. Testes were obtained immediately following euthanasia by CO_2_ asphyxiation, fixed in 4% paraformaldehyde, embedded in paraffin and obtained paraffin sections at 5 μm with a microtome. Frozen sections at 8 μm obtained in a cryostat at -22°C were fixed in 4% PFA for 10 min. IF was performed using either the FITC or TRITC-conjugated secondary antibodies (Jackson ImmunoResearch, West Grove, PA). For IHC, tissue sections were de-paraffinized and rehydrated, to be followed by antigen retrieval in 10 mM sodium citrate buffer for 15 min. Positive staining was visualized using DAB substrate kits (Zhong Shan Technology, China) and sections were counterstained with hematoxylin. Antibodies were obtained commercially as follows: Ki67 (1:1000, ab15580, Abcam), α-SMA (1:400, S0010/ab137734, Epitomics/Abcam), PCNA (1:100, 2586, Cell Signaling Technology), HSD3B1 (1:400, sc-30820, Santa Cruz), CYP11A1 (1:500, AB1244, Chemicon/Millipore), VCAM1 (1:400; AF643; R&D) and JAG1 (1:50, sc-6011, Santa Cruz). IF and IHC was performed as described [15, 16]. Images were examined and acquired using a Nikon Eclipse 80i fluorescence microscope (Tokyo, Japan) with a built-in Nikon CCD camera for image acquisition. Image overlays and relative fluorescence intensity was quantified using Image J software.

### Quantitative analysis of peritubular myoid cells (PMCs), fetal Leydig cells (FLCs), vascular smooth muscle cell (VSMCs) and interstitial progenitor cells

Paraffin or frozen sections were used for dual-labeled immunofluorescence analysis with antibodies specific for PMCs or VSMC (e.g., α-SMA with PCNA served as a proliferation marker), FLCs (e.g., HSD3B1 and CYP11A1 with Ki67 served as a proliferation marker), and interstitial progenitor cells (e.g., VCAM1). Cell nuclei were visualized by DAPI. Percentage of PCNA-positive (PCNA^+^) or Ki67-positive (Ki67^+^) cells was used to illustrate the mitotic index of PMCs and FLCs, the ratio of PMCs:FLCs and interstitial progenitor cells:FLCs was used to assess the differentiated status of interstitium. Cells were counted using 6–12 randomly selected images with at least ~760 PMCs/testis in α-SMA/PCNA, ~230 VSMCs/testis in α-SMA/PCNA, ~550 FLCs/testis in HSD3B1/Ki67, ~780:280 PMCs:FLCs/testis in α-SMA/HSD3B1, and ~330:160 interstitial progenitor cells:FLCs/testis in VCAM1/CYP11A1, mouse group with n = 3 mice in each group for analysis.

### RNA extraction, reverse transcription (RT) and quantitative real time polymerase chain reaction (qPCR)

Total RNA was extracted from the whole testes using RNeasy kits (Promega) according to the manufacturer’s instructions. Total RNA (1–2 μg) was reverse transcribed in a final volume of 20 μl using oligo-dT and M-MLV Reverse Transcriptase kit (Promega). To quantify the steady-state mRNA level of a target gene, real-time SYBRGreen assay was performed using cDNAs obtained in the RT step relative to the expression of the house-keeping gene *Gapdh* (glyceraldehyde-3-phosphate dehydrogenase). Primers used for the RT-PCR are listed in **S1 Table**. The authenticity of PCR products was confirmed by direct nucleotide sequencing.

### Western Blot Analysis

Western blot analysis was performed as described [15]. Fragments of testes were lysed in radio-immunoprecipitation assay lysis buffer (RIPA) containing Complete Mini Protease Inhibitor Cocktail Tablets (Roche). Protein concentration in the supernatant was estimated using the Bradford assay (Bio-Rad Laboratories). About 40 μg protein per lane was used for immunoblotting under reducing conditions using 12% SDS-containing polyacrylamide gels using corresponding primary antibody: α-SMA (1:2000, S0010/ab137734, Epitomics/Abcam), HSD3B1 (1:1000, sc-30820, Santa Cruz), CYP11A1 (1:2000, AB1244, Chemicon/Millipore), VCAM1 (1:2000; AF643; R&D), JAG1 (1:1000, sc-6011, Santa Cruz) and β-TUBULIN (1:3000, E7, Developmental Studies Hybridoma Bank, Iowa City, IA), to be followed by an incubation with an Odyssey IRDye 680CW (red) or 800CW (green) secondary antibody (1:20000; LI-COR Bioscience) for 1 hour at room temperature. Specific signals and corresponding protein band intensities were evaluated using an Odyssey Infrared Imaging system and software (Version 3.0).

### Statistical analysis

Experiments were repeated at least three times using different mice or cultures. Data were evaluated for statistical differences using Student*’s t*-test. Differences were considered significant with a *P* value of <0.05.

## Results

### Sertoli cell-specific deletion of *Wt1* perturbs peritubular myoid cell (PMC) differentiation during fetal testis development

**Fig 1:**
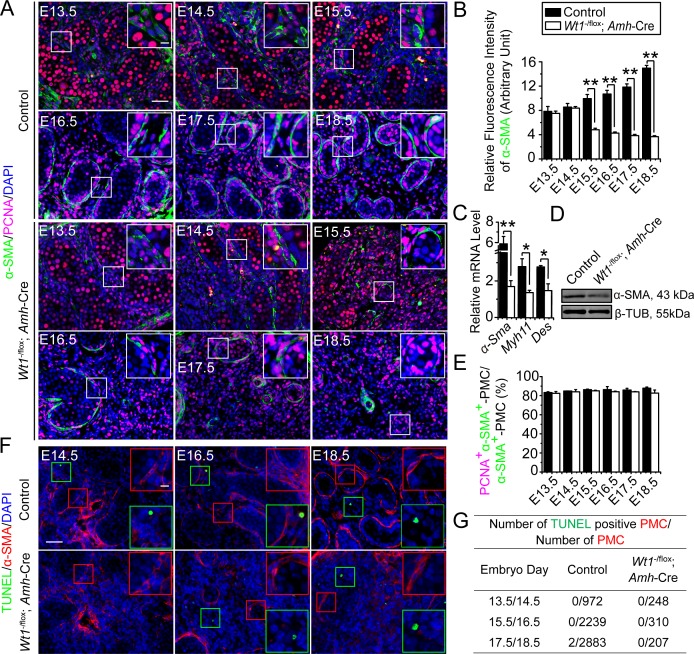
Status of peritubular myoid cells in *Wt1*^SC-cKO^ testis during fetal mouse testis development. **(A)** Immunofluorescence analysis of peritubular myoid cell (PMC) marker α-SMA (FITC, green fluorescence), and proliferation marker PCNA (TRITC, red fluorescence) in cross-sections of control *vs*. *Wt1*^SC-cKO^ mouse testes in E13.5 to E18.5. Insets are the corresponding magnified views of the boxed areas. In control testes, α-SMA stained spindle-shaped PMCs. In E13.5 to E15.5 fetal testes, interstitial cells including interstitial progenitor cells were positive for α-SMA. However, the α-SMA expression was strongly up-regulated in PMCs and down-regulated in the interstitial cells in E16.5 to E18.5 fetal testes. The number of PMCs increased during fetal testis cord assembly from E13.5 to E18.5, in addition, most of the α-SMA-positive (α-SMA^+^) cells were in active cell cycles which were positive for PCNA. In *Wt1*^SC-cKO^ testes, the number of α-SMA^+^ cells were down-regulated from E16.5 to E18.5, however, the α-SMA^+^ cells remained highly proliferative which were positive for PCNA. **(B)** α-SMA relative fluorescence intensity was used to estimate PMC differentiation status in E13.5 to E18.5 control and *Wt1*^SC-cKO^ testes. The fluorescence intensity increased in E15.5 to E18.5 in control testes but reduced in *Wt1*^SC-cKO^ testes, illustrating *Wt1* deletion led to a decrease in PMC differentiation. **(C)** qPCR analysis of PMC marker genes *α-Sma*, *Myh11* and *Des* in *Wt1*^SC-cKO^ mouse testes *vs*. controls in postnatal day 1 (P1). **(D)** Immunoblot analysis of the PMC-associated protein α-SMA in *Wt1*^SC-cKO^ mouse testes *vs*. controls in P1. The steady-state level of α-SMA proteins in P1 mutant testes was significantly down-regulated. **(E)** Mitotic index expressed as the ratio of PCNA^+^ and α-SMA^+^ PMCs to total PMCs (PCNA^+^ α-SMA^+^ PMCs:α-SMA^+^ PMCs). PMC mitotic indexes did not change between control and *Wt1*^SC-cKO^ testes from E13.5 to E18.5. **(F)** PMC marker α-SMA (red fluorescence) and apoptotic signals (TUNEL assay) in E14.5 to E18.5 control and *Wt1*^SC-cKO^ testes were assessed. Insets are the corresponding magnified views of the colored boxed areas. TUNEL^+^ (green insets) did not overlay with α-SMA^+^ PMCs in all ages examined (red insets). **(G)** Effects of Sertoli cell-specific deletion of *Wt1* on PMC apoptosis. E, embryonic day; scale bar = 50 μm, and 10 μm in inset, which applies to all micrographs. Each bar is a mean±SEM of *n* = 3 mice. *, *P*<0.05; **, *P*<0.01.

We used Sertoli cell expressed *Amh*-Cre transgene, the *Wt1*-null allele (*Wt1*^+/−^) and the *Wt1*^flox^ mouse strains to obtain Sertoli cell-specific *Wt1* ablation in testes of *Wt1*^- /flox^; *Amh*-Cre males (i.e., *Wt1*^SC-cKO^ mice) at E14.5. It was reported that Sertoli cell-specific deletion of *Wt1* disrupted testis cord formation in fetal testes [11], and PMCs were shown to work cooperatively with Sertoli cells to assemble functional testis cords [7]. To assess if *Wt1* deletion-induced failure in testis cord formation is mediated by perturbing the differentiation and proliferation of PMCs, we used PMC marker α-SMA, and proliferative marker PCNA for dual-labeled immunofluorescence analysis and quantification to assess the status of PMCs **(Figs 1A, S1 and S2)**. In control testes, α-SMA-positive (α-SMA^+^) PMCs were properly differentiated, and they remained mitotically active to support the assembly of testis cords from E13.5 to E18.5 **(Figs 1A, S1 and S2)**. α-SMA relative fluorescence intensity, an indicator of PMC differentiation status, was induced from E13.5 to E18.5 **(Fig 1B)**, illustrating the differentiation of PMCs during fetal testis development. In *Wt1*^SC-cKO^ testes, we observed a considerably reduction in the number of α-SMA^+^ PMCs from E15.5 in the remnant testis cords **(Fig 1A)**, consistent with the declining α-SMA relative average fluorescence intensity from E15.5 **(Fig 1B)**, and mRNA **(Fig 1C)** or protein **(Fig 1D)** levels of PMC markers (mRNA: *α-Sma*, *Myh11* and *Des*; protein: α-SMA) in postnatal day 1 (P1) *Wt1*^SC-cKO^ testes *vs*. control testes. Interestingly, PMCs were found to be mitotically active **(Fig 1A)**, and the ratios of mitotically active PMCs (expressed as PCNA^+^ and α-SMA^+^ PMCs: α-SMA^+^ PMCs) were not altered **(Fig 1E)** from E14.5 to E18.5 between the mutant and control groups. We also observed no significant changes in apoptotic status of these cells between the mutant and the control groups **(Figs 1F, 1G and S3)** indicating changes in the numbers of PMCs in *Wt1*^SC-cKO^ *vs*. control testes were likely the result of a reduction in PMC differentiation. These results indicate that Sertoli cell *Wt1* is involved in PMC differentiation, thereby supporting testis cord assembly.

### Sertoli cell-specific deletion of *Wt1* in *Wt1*^SC-cKO^ mutant mice perturbs the differentiation and proliferation of fetal Leydig cells (FLCs)

**Fig 2:**
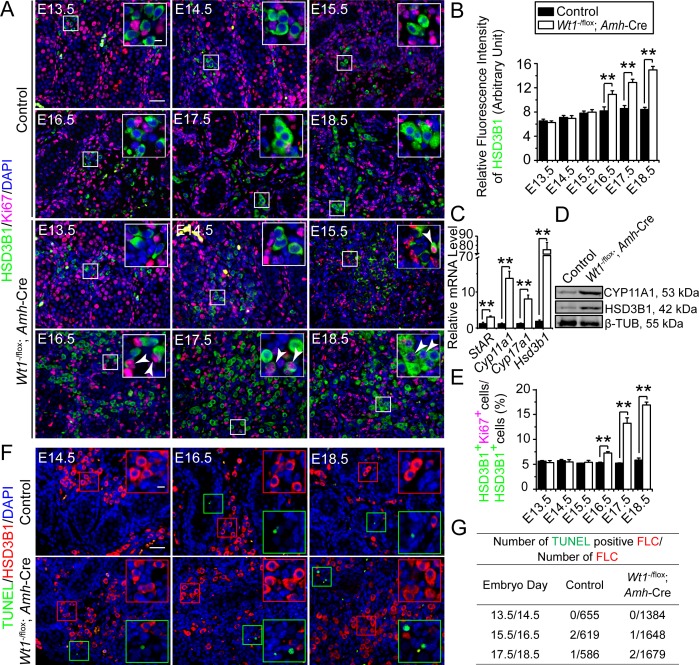
Fetal Leydig cells (FLCs) differentiate, but having abnormal proliferation status in the interstitium of *Wt1*^SC-cKO^ mouse testes during fetal testis development. **(A)** Immunofluorescence analysis of FLC marker HSD3B1 (FITC, green fluorescence) and proliferation marker Ki67 (TRITC, red fluorescence) in cross-sections of control *vs*. *Wt1*^SC-cKO^ mouse testes in E13.5 to E18.5. Insets are the corresponding magnified views of the boxed areas. FLCs found in the testicular interstitium were rarely mitotically active in control testes, while in mutant testes FLC density was gradually increased and some FLCs remained mitotically active (white arrowheads) from E16.5 to E18.5. **(B)** HSD3B1 relative fluorescence intensity was used to estimate FLC differentiation status in E13.5 to E18.5 control and *Wt1*^SC-cKO^ testes. The fluorescence intensity increased in E13.5 to E16.5 and maintained in E16.5 to E18.5 in control testes but increased in E13.5 to E18.5 in *Wt1*^SC-cKO^ testes, illustrating *Wt1* deletion led to an increase in FLC differentiation. **(C)** qPCR analysis of FLC marker genes *StAR*, *Cyp11a1*, *Cyp17a1*, *Hsd3b1* in *Wt1*^SC-cKO^ mouse testes *vs*. controls in P1. **(D)** Immunoblot analysis of the FLC-associated protein CYP11A1 and HSD3B1 in *Wt1*^SC-cKO^ mouse testes *vs*. controls in P1. The steady-state level of CYP11A1 and HSD3B1 proteins in P1 *Wt1*^SC-cKO^ testes was considerably up-regulated. **(E)** Mitotic index expressed as the ratio of HSD3B1-positive and Ki67-positive cells to HSD3B1-positive cells (HSD3B1^+^ Ki67^+^ cells:HSD3B1^+^ cells) was used to estimate mitotic activity of FLC in E13.5 to E18.5. FLCs in control testes were less proliferative, however, the mitotic index of FLC in mutant testes was significantly up-regulated from E16.5 to E18.5. **(F)** FLC marker HSD3B1 (red fluorescence) and apoptotic signals (TUNEL assay) in E14.5 to E18.5 control and *Wt1*^SC-cKO^ testes were assessed. Insets are the corresponding magnified views of the colored boxed areas. TUNEL^+^ (green insets) did not overlay with HSD3B1^+^ cells in all ages examined (red insets). **(G)** Effects of Sertoli cell-specific deletion of *Wt1* on FLC apoptosis. E, embryonic day. Scale bar = 50 μm, and 10 μm in inset, which applies to all micrographs. Each bar is a mean±SEM of *n* = 3 mice. **, *P*<0.01.

FLCs were reported to regulate testis cord elongation and expansion [6]. In order to assess whether *Wt1* ablation disrupted testis cord formation was mediated through a disruption of FLC development during fetal testis compartmentalization, we performed dual-labeled immunofluorescence to monitor FLC differentiation, and assessed the HSD3B1 relative fluorescence intensity and ratio of mitotically active FLC expressed as HSD3B1:Ki67 **(Figs 2A, S4 and S5)**. In control testes, FLCs differentiated and progressively arranged in clusters in fetal testes from E13.5-E18.5 **(Figs 2A, S4 and S5)**. HSD3B1 relative fluorescence intensity increased from E13.5 to E16.5, and maintained in E16.5 to E18.5 **(Fig 2B),** illustrating the differentiation of FLCs in the early embryo day (e.g., from E13.5 to E15.5) and involution in the late embryo day (e.g., from E15.5 to E18.5). In *Wt1*^SC-cKO^ testes, FLCs also differentiated normally, however, FLC density was induced and progressively developed into larger clusters from E16.5 **(Figs 2A, S4 and S5)**. Consistent with these findings, we also detected an up-regulation on HSD3B1 relative fluorescence intensity from E16.5 **(Fig 2B)**, and the expression of several FLC marker genes *StAR*, *Cyp11a1*, *Cyp17a1* and *Hsd3b1* by qPCR **(Fig 2C)**, and also CYP11A1 and HSD3B1 by immunoblotting in P1 *Wt1*^SC-cKO^ whole testes *vs*. control testes **(Fig 2D)**. Also, in control testes, FLCs were mitotically inactive in fetal testes from E13.5-E18.5 **(Fig 2A)**, supporting the notion that FLCs proliferate by recruitment from a precursor cell pool instead of via mitotic division of differentiated FLCs [17, 18]. Yet FLCs in *Wt1*^SC-cKO^ testes were mitotically active **(Fig 2A)** and their mitotic indexes **(Fig 2E)** increased considerably from E16.5 to E18.5 indicating Sertoli cell *Wt1* modulated FLC differentiation and proliferation in fetal testes. We detected no difference in apoptosis of FLCs by TUNEL assay in *Wt1*^SC-cKO^ *vs*. control testes **(Figs 2F, 2G and S6)**. These results suggest that Sertoli cell *Wt1* regulated FLC differentiation and proliferation, which in turn modulated testis cord assembly.

### Sertoli cell-specific deletion of *Wt1* disrupts differentiation status of FLC, PMC and interstitial progenitor cell pools

**Fig 3:**
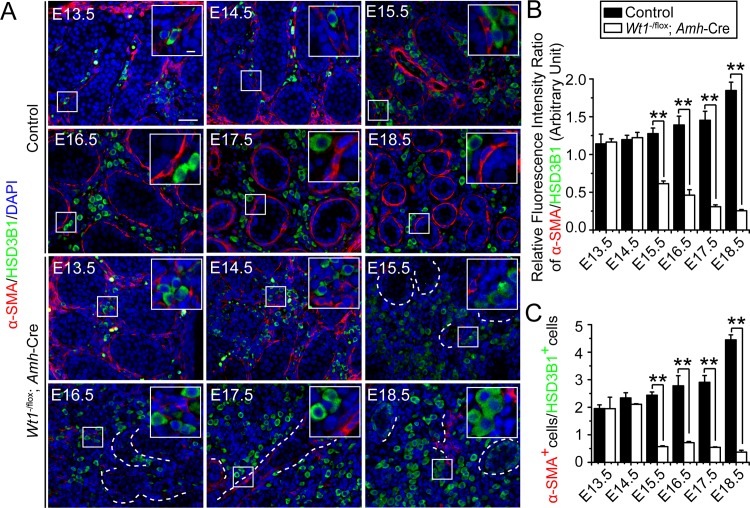
Disruption on the differentiation status of PMC and FLC in *Wt1*^SC-cKO^ mice that leads to a reduced ratio of PMC:FLC during fetal testis development. **(A)** Immunofluorescence analysis of PMC marker α-SMA (TRITC, red fluorescence) and HSD3B1 (FITC, green fluorescence) in cross-sections of control *vs*. *Wt1*^SC-cKO^ mouse testes from E13.5 to E18.5. Insets are the corresponding magnified views of the boxed areas. In control testes, the number of α-SMA^+^ PMCs increased considerably from E13.5 to E18.5 during fetal testis assembly. HSD3B1-stained FLCs were found between testis cords during the development of interstitium. In *Wt1*^SC-cKO^ testes, deletion of *Wt1* in Sertoli cells led to a considerable reduction in PMC number from E15.5 to E18.5. HSD3B1-positive (HSD3B1^+^) FLCs were found to be differentiated, forming cell clusters from E16.5 to E18.5 in *Wt1*^SC-cKO^ testes. The α-SMA^+^ PMCs were found in and around the remnant tubules, and normal testis architecture was not established. **(B)** The ratio of α-SMA to HSD3B1 relative fluorescence intensity was obtained by measuring the relative fluorescence intensity, which increased in E15.5 to E18.5 in control testes but reduced in *Wt1*^SC-cKO^ testes, illustrating Wt1 deletion led to changes in the differentiation status of PMC *vs*. FLC. **(C)** Consistent with findings shown in **(B)**, the ratio of α-SMA^+^ PMCs:HSD3B1^+^ FLCs (α-SMA^+^ PMCs:HSD3B1^+^ FLCs) which obtained by scoring these two cell types increased in E15.5 to E18.5 in control testes but reduced in *Wt1*^SC-cKO^ testes.

**Fig 4:**
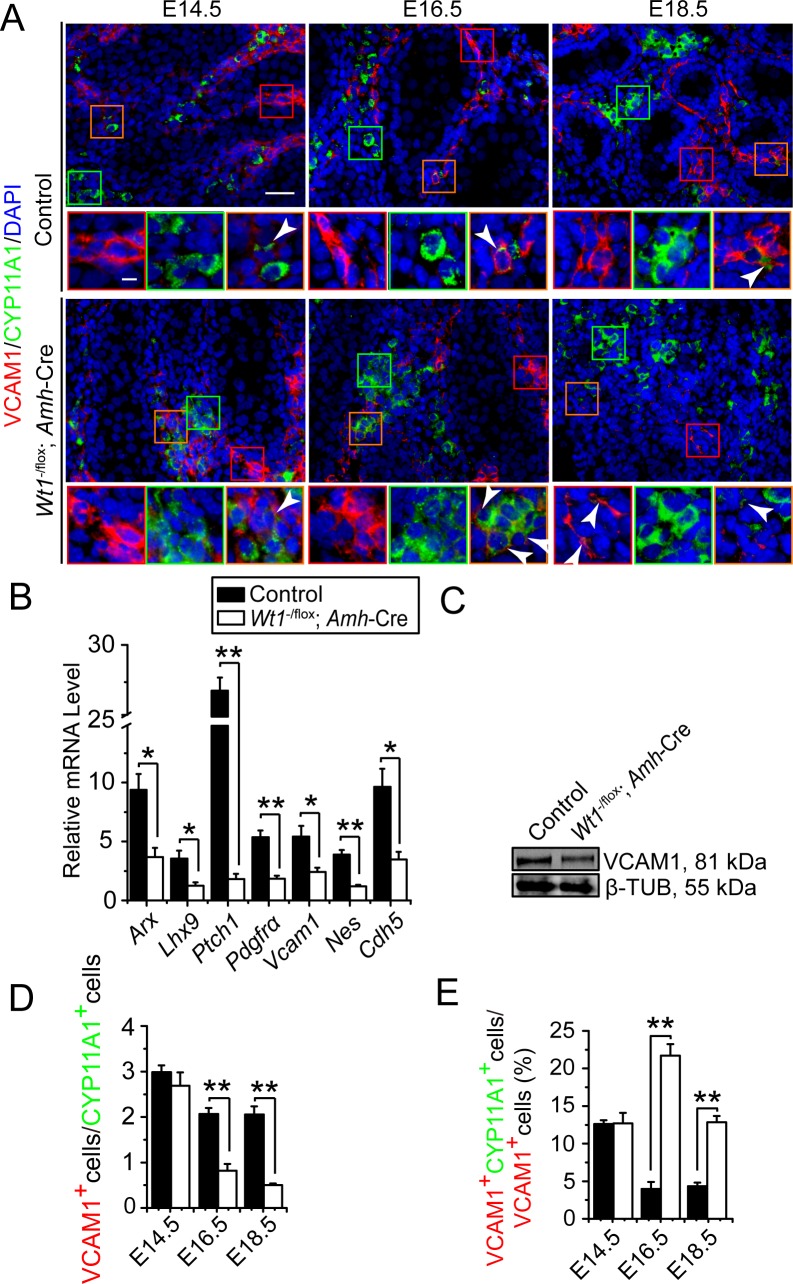
Uncharacterized interstitial progenitor cells are considerably differentiated to FLCs in *Wt1*^SC-cKO^ *vs*. control testes during fetal testis development. **(A)** Immunofluorescence analysis of uncharacterized interstitial progenitor cell marker VCAM1 (TRITC, red fluorescence), and FLC marker CYP11A1 (FITC, green fluorescence) in cross-sections of control *vs*. *Wt1*^SC-cKO^ mouse testes in E14.5 to E18.5. Insets are the corresponding magnified views of the colored boxed areas. In control testes, VCAM1 stained testicular undifferentiated interstitial progenitor cells which contained FLC precursor cells (red box). CYP11A1 stained FLCs which were present between cords (green box). VCAM1 and CYP11A1 double stained early differentiated FLCs (orange box, arrow heads). In *Wt1*^SC-cKO^ mouse testes, uncharacterized interstitial progenitor cells were found near the remnant cords (red box), and FLCs formed dense clusters in the testicular interstitium (green box). Clusters of early differentiated FLCs were found in E16.5 *Wt1*^SC-cKO^ mouse testes (orange box, white arrow heads). **(B)** qPCR analysis of interstitial progenitor cell marker genes *Arx*, *Lhx9*, *Patched1*, *Pdgfα*, *Vcam1*, *Nestin*, *Cdh5* in *Wt1*^SC-cKO^ mouse testes *vs*. controls in P1. **(C)** Western blot analysis of the interstitial progenitor cell-associated protein VCAM1 in *Wt1*^SC-cKO^ mouse testes *vs*. controls in P1. The steady-state levels of VCAM1 protein was significantly down-regulated. **(D)** Testicular interstitial progenitor cell relative index to FLC expressed as the ratio of VCAM1-positive (VCAM1^+^) interstitial cells to CYP11A1-positive (CYP11A1^+^) FLCs (VCAM1^+^ cells:CYP11A1^+^ cells) was used to estimate the balance of interstitial progenitor cell maintenance and FLC differentiation during fetal testis development. The ratio of interstitial progenitor cells to FLCs was down-regulated in *Wt1*^SC-cKO^ mouse testes from E16.5 to E18.5. **(E)** FLC differentiation index, expressed as the ratio of VCAM1^+^ CYP11A1^+^ cells to VCAM1^+^ cells (VCAM1^+^ CYP11A1^+^ cells:VCAM1^+^ cells), was used to estimate the differentiation ratio of interstitial progenitor cells to FLCs. FLC differentiation index was increased in *Wt1*^SC-cKO^ mouse testes from E16.5 to E18.5. E, embryonic day; scale bar = 50 μm, and 10 μm in inset, which applies to all micrographs. Each bar is a mean±SEM of *n* = 3 mice. *, *P*<0.05; **, *P*<0.01.

Some of the FLCs and PMCs are proposed to be originated from a common precursor cell pool, namely the testis interstitial progenitor cells [19–22]. In order to assess whether *Wt1* ablation disrupted the differentiation status of PMC *versus* FLC during fetal testis development, we performed dual-labeled immunofluorescence to monitor spatiotemporal expression of PMC marker α-SMA and FLC marker HSD3B1 **(Figs 3A, S7 and S8)**. We also compared the relative differentiation status of PMC and FLC by assessing the ratio of HSD3B1: α-SMA relative fluorescence intensity **(Fig 3B)** and α-SMA positive PMCs:HSD3B1 positive FLCs (α-SMA^+^ PMCs:HSD3B1^+^ FLCs) **(Fig 3C)**. In control testes, the HSD3B1: α-SMA relative fluorescence intensity ratio and α-SMA^+^ PMCs:HSD3B1^+^ FLCs ratio increased from E13.5 to E18.5 **(Fig 3B and 3C)**. However, both ratios reduced in *Wt1*^SC-cKO^ testes from E15.5 to E18.5 **(Fig 3B and 3C)**, indicating the differentiation status of PMCs *versus* FLCs was perturbed after Sertoli cell *Wt1* ablation during fetal testis development. In order to assess whether *Wt1* ablation perturbed the differentiation of interstitial progenitor cell pools, we further analyzed the differentiation status of fetal testis interstitial progenitor cells by dual-labeled immunofluorescence of interstitial progenitor cell marker VCAM1 and FLC marker CYP11A1. In control testes, we observed some VCAM1-positive (VCAM1^+^) and CYP11A1-positive (CYP11A1^+^) interstitial cells in the interstitium, supporting the notion that FLCs were differentiated from interstitial progenitor cells **(Figs 4A and S9)**. In *Wt1*^SC-cKO^ testes, the number of VCAM1^+^ interstitial progenitor cells was found to be reduced in E18.5 testes **(Figs 4A and S9)**, as well as the expression of interstitial progenitor cell marker genes *Arx*, *Lhx9*, *Patched1*, *Pdgfrα*, *Vcam1*, *Nestin*, *Cdh5* **(Fig 4B)**, and VCAM1 proteins in P1 *Wt1*^SC-cKO^ testes **(Fig 4C)**. The interstitial progenitor cell (VCAM1^+^ cells):FLC (CYP11A1^+^ cells) ratios were also considerably reduced **(Fig 4D)**, whereas the newly differentiated FLCs (VCAM1^+^ CYP11A1^+^ cells):FLC progenitor cells (VCAM1^+^ cells) ratios were induced **(Fig 4E)** at E16.5 and E18.5. Collectively, these findings illustrated that the ratio of PMC:FLC and interstitial progenitor cell:FLC were significantly altered, and more FLCs were found to be differentiated from the progenitor cell pool during fetal testis development in *Wt1*^SC-cKO^ mice.

### Vasculature is formed but Notch signaling is down-regulated in *Wt1*^SC-cKO^ mutant mice during fetal testis development

**Fig 5:**
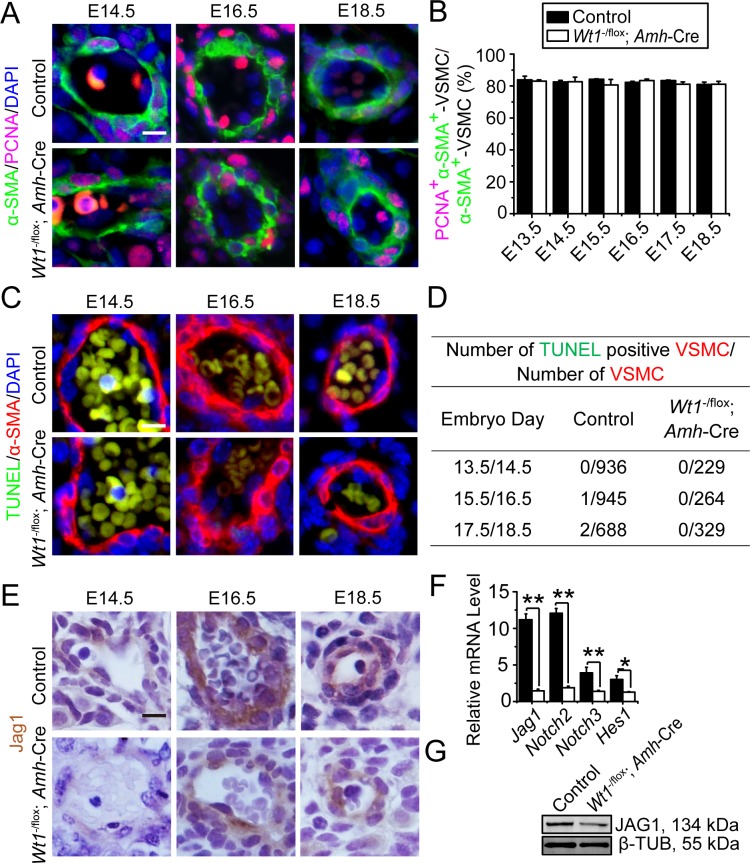
Normal vasculature development but down-regulation of Notch signaling are found in *Wt1*^SC-cKO^ *vs*. control testes during fetal testis development. **(A)** Immunofluorescence analysis of vascular smooth muscle cell (VSMC) marker α-SMA (FITC, green fluorescence), and proliferation marker PCNA (TRITC, red fluorescence) in cross-sections of control *vs*. *Wt1*^SC-cKO^ mouse testes in E14.5, E16.5 and E18.5. Insets are the corresponding magnified views of the boxed areas. VSMCs were normally differentiated and proper vasculature was established in both control and *Wt1*^SC-cKO^ testes during fetal testis morphogenesis. **(B)** Mitotic index expressed as the ratio of PCNA^+^ α-SMA^+^ VSMCs to total VSMCs (PCNA^+^ α-SMA^+^ VSMCs:α-SMA^+^ VSMCs) was used to estimate mitotic activity of VSMC. VSMC mitotic index did not change between control and *Wt1*^SC-cKO^ testes from E13.5 to E18.5. **(C)** VSMC marker α-SMA (red fluorescence) and apoptotic signals (TUNEL assay) in E14.5, E16.5 and E18.5 control and *Wt1*^SC-cKO^ testes were assessed. Insets are the corresponding magnified views of the colored boxed areas. TUNEL^+^ (green insets) did not overlay with α-SMA^+^ VSMCs in all ages examined (red insets). **(D)** Effects of SC-specific deletion of *Wt1* on VSMC apoptosis. **(E)** Immunohistochemistry analysis of Notch signaling ligand JAG1 in cross-sections of control *vs*. *Wt1*^SC-cKO^ mouse testes in E14.5, E16.5 and E18.5. JAG1 was expressed in vasculature-associated interstitial cells in control testes. The JAG1 expression level was down-regulated comparing to corresponding controls in *Wt1*^SC-cKO^ mouse testes in E14.5, E16.5 and E18.5. **(F)** qPCR analysis of Notch ligand gene *Jag1*, receptor genes *Notch2*, *Notch3*, target gene *Hes1* in *Wt1*^SC-cKO^ mouse testes *vs*. controls in P1. **(G)** Western blot analysis of Notch signaling ligand protein JAG1 in *Wt1*^SC-cKO^ mouse testes *vs*. controls in P1. The steady-state levels of JAG1 protein was significantly down-regulated. SE, embryonic day; scale bar = 50 μm, and 10 μm in inset, which applies to all micrographs. Each bar is a mean±SEM of *n* = 3 mice. *, *P*<0.05; **, *P*<0.01.

Genetic studies have shown that Notch signaling restricts FLC differentiation to maintain a balance between differentiated FLC and their progenitor cells during fetal testis development [23, 24]. An activation of Notch signaling was detected in vasculature and vasculature-associated interstitial cells during fetal testis development [24]. Vasculature is known to be involved in testis cord formation and fetal testis morphogenesis [25–28]. In order to assess whether deletion of *Wt1* in Sertoli cells would impede vasculature development, we used dual-labeled immunofluorescence analysis by utilizing vascular smooth muscle cell (VSMC) marker α-SMA and proliferation marker Ki67 to examine vascular development in the fetal testis. Normal differentiation, proliferation and apoptosis of VSMCs were noted in *Wt1*^SC-cKO^ mouse testes, illustrating that the deletion of *Wt1* had no impact on interstitial vasculature development in *Wt1*^SC-cKO^ mouse testes **(Figs 5A–5D, S10 and S11)**. We next investigated changes of Notch signaling in control and *Wt1*^SC-cKO^ testes. In control testis, Notch signaling ligand JAG1 was expressed in vasculature-associated interstitial cells **(Fig 5E)**, in consistent with previous study [24]. In *Wt1*^SC-cKO^ testes, the expression of JAG1 was reduced when compared to corresponding controls in E14.5, E16.5 and E18.5 testes **(Fig 5E)**. We also detected considerable down-regulations of Notch signaling ligand gene *Jag1*, receptor genes *Notch2* and *Notch3*, the downstream target gene *Hes1* **(Fig 5F)**, and the JAG1 protein in P1 *Wt1*^SC-cKO^ testes **(Fig 5G)**, illustrating that *Wt1* ablation resulted in down-regulating Notch signaling in fetal testes. The down-regulation of Notch signaling, in turn, failed to maintain the balance between FLC differentiation and interstitial progenitor cell preservation in which more interstitial progenitor cells were found to differentiate to FLCs. In summary, our results illustrated that *Wt1* modulated FLC differentiation and interstitial progenitor cell pool maintenance through Notch signaling in the developing fetal testis.

## Discussion

During embryonic development, organization of the testis is a complex cellular process since several cell lineages are involved in forming a testis *vs*. an ovary. In XY male embryos, SRY serves as a molecular switch in pre-Sertoli cells at around embryo day 10.5 (E10.5) to stimulate Sertoli cell specification, proliferation and testis cord assembly to develop into the testis [reviewed in [4, 29]]. Testis cell proliferation and cord assembly are the morphological hallmarks of fetal testis development vs. the ovary. Although Sertoli cells are the major driving force in testis cord formation, peritubular myoid cells (PMCs) and fetal Leydig cells (FLCs) are reported to be critical in testis cord assembly and interstitium compartmentalization as well [6, 7]. Previous studies have shown that *Wt1* maintains gonad development [10], testicular cord integrity [11, 12], Sertoli cell specification [13] and dictates the fate of FLCs [15] in fetal testis development. Herein, we provided new insights into the role of *Wt1* in fetal testis compartmentalization by modulating PMC and FLC differentiation and proliferation.

Mouse testis cord assembly initiates at E11.5-E12.5 day, associated with the differentiation of Sertoli cells at E10.5, FLCs at E12.5 and PMCs at E13.5 [reviewed in [29]]. However, Sertoli cell-specific deletion of *Wt1* using *Amh*-Cre takes place at E13.5-E14.5 [11], illustrating *Wt1* deletion occurs shortly after the initiation of the differentiation of Sertoli cells, FLCs and PMCs, and testis cord assembly. Thus, some remnant features of testis cords, residual Sertoli cells and PMCs were detected in and around these cords and FLCs were also detected in fetal *Wt1*^SC-cKO^ mouse testes before the activity of *Amh*-Cre. PMC abnormalities are uncommon during fetal testis development. *Dhh* (a Sertoli cell marker gene) knockout [30, 31] and Sertoli cell ablation mice [32, 33] display compromised PMC differentiation in the developing testes suggesting the involvement of Sertoli cell in PMC differentiation and function. Thus, Sertoli cell transcription factor *Wt1* may directly control PMC differentiation and function. The known functions ascribed to PMCs in the testis include seminiferous tubular contraction and sperm transport in adult animals [34]. Studies have shown that PMCs also secrete a number of biomolecules, such as peritubular factors that modulate Sertoli cell function (PModS) [35], fibroblast growth factor 2 (FGF2) [36], colony stimulating factor 1 (CSF1) [37], and Insulin-like growth factor 1 (IGF1) [36], which thus modulate testis microenvironment in adult animals. PMC was also found to involve in testicular growth in postnatal rat testis [38] and spermatogenesis through androgen signaling [39, 40], Lgr4-Wnt/β-catenin signaling [41] and glial cell line-derived neurotrophic factor (GDNF) [40, 42] in adult mouse testis. However, little is known about the function of PMC during fetal testis development. During testis cord assembly, Sertoli cells produce and deposit collagen IV and small amounts of laminin, whereas PMCs secrete fibronectin, collagen I and IV to form the basement membrane that surrounds the testis cords [7]. The intimate interaction and communication between PMCs and Sertoli cells are required for the formation of basement membrane during fetal testis development. Therefore, the aberrant PMC development further contributed to the testis cord disruption in *Wt1*^SC-cKO^ fetal testes. However, the function of PMC and Sertoli cell-PMC interactions during fetal testis development remain largely unknown.

The failure of fetal testis cord assembly is known to cause aberrant Leydig cell development in the interstitium [23, 24, 43–45]. On the other hand, FLCs differentiate at ~E12.5 in fetal mouse testis also play an active role in fetal testis cord assembly [6]. Deletion of *Wt1* led to a down-regulation of *Dhh* and *platelet-derived growth factor-α (Pdgfα*) [15], which are Sertoli cell-derived paracrine factors that coordinate FLC lineage differentiation but have limited effects on the maintenance and/or expansion of FLC population [46, 47]. However, FLCs were found in the interstitium of fetal *Wt1*^SC-cKO^ mouse testes, illustrating FLC lineage was maintained even when Sertoli cells had lost their identities [11, 13]. These findings suggest that once the fate of FLC progenitors is determined, its development course would continue regardless of the Sertoli cell fate. We also observed abnormal differentiation and proliferation of FLCs in *Wt1*^SC-cKO^ mouse during fetal testis assembly. Although the proliferation and differentiation of FLCs from interstitial progenitor cells remained consistently high at around E16.5 to E18.5 in *Wt1*^SC-cKO^ mouse *vs*. controls, the high differentiation rate and the decrease of VCAM1^+^ interstitial progenitor cells also indicated the exhaustion of interstitial progenitor cells. Thus, the total FLC number was not affected in postnatal day 1 *Wt1*^SC-cKO^ mouse *vs*. controls [15]. The origin of PMC remains unknown, however, and PMC is not likely derived from migrating mesonephric cells [20, 21]. Since *Dax1* [48] and *Dhh* [30, 31, 46] knockout mice all displayed a pattern of disrupted FLC and PMC development, seemingly suggest that FLCs and PMCs may have originated from a common precursor, namely interstitial progenitor cells. Furthermore, PMCs express many genes in common with interstitial cells in fetal testis development [22] that PMCs likely originate from interstitial progenitor cells. The continual differentiation of interstitial progenitor pools to FLCs thus impeded subsequent PMC population when the interstitial progenitor cell pool was considerably reduced due to their over commitment to differentiate into FLCs, thereby perturbing the proper ratio of FLC:PMC in the mutant mouse testis. In addition, FLCs and Sertoli cells are likely sharing some of the progenitor cells from coelomic epithelium [reviewed in [4]]. *Wt1* is known to be involved in the maintenance of Sertoli cell lineage, thus deletion of *Wt1* causes a reprogramming of Sertoli cells by committing to become Leydig-like cells, leading to Sertoli cell-to-FLC trans-differentiation and thereby fetal testis cord disruption in *Wt1*^SC-cKO^ mouse testes [13]. It is possible that some of the FLCs observed in our studies were in fact Sertoli cells that trans-differentiated from Sertoli cells. However, it is noted that only a small number of Leydig-like Sertoli cells survived in adult testes as earlier reported [13], indicating such Sertoli cell tras-differentiation Leydig-like cells are a minority in *Wt1*^SC-cKO^ mouse testes. Taken collectively, these data suggest that the failure of testis cord assembly in *Wt1*^SC-cKO^ testes is contributed by the aberrant differentiation and development of Sertoli cells as previous reported [11, 13], and also by the aberrant differentiation of PMCs, coupled with the imbalanced interstitial progenitor cell and FLC development as reported herein. Furthermore, the homeostasis of testis morphogenesis is likely maintained through proper signaling among Sertoli cells, PMCs and FLCs.

Leydig cell development in the mouse testis exhibits a distinctive bi-phase pattern, with rapid expansion of FLC population and adult Leydig cell (ALC) population at two different developmental periods. First, FLCs appear in fetal testes by embryonic day 12.5 (E12.5), which in turn undergo gradual atrophy (also known as involution or degeneration) during postnatal development. Second, ALCs, which are originated from their own progenitor cell pool instead of FLCs, appear around postnatal day 7 to 10 testes, which gradually replace FLCs by postnatal 2 to 3 weeks (for reviews, see [49, 50]). In a recent report from our laboratory [15], FLCs are persistently found in adult testes that form FLC-like cell clusters, which dominate the entire adult testis instead of ALCs when *Wt1* was deleted from Sertoli cells in fetal testes. Herein, we report findings in which *Wt1* was shown to regulate FLC differentiation through over commitment of interstitial progenitor cells to differentiate into FLCs, which is modulated via a down-regulation of Notch signaling in *Wt1*^SC-cKO^ mouse testes. Because ALC progenitor cells are present in fetal testes [51], the interstitial progenitor cells in fetal testes may also include ALC progenitor cells. As such, over commitment of interstitial progenitor cells to form FLCs in fetal testes thus impedes subsequent ALC differentiation in postnatal testes. Taken together, *Wt1* likely maintain an intriguing balance between the interstitial progenitor cell pool and FLC differentiation in fetal testes. *Wt1* also plays a role in maintaining the ALC population in postnatal testes probably through its maintenance on ALC progenitor cell pool in fetal period.

Vasculature-interstitial cell crosstalk through Vegf signaling [25, 26], vasculature reorganization mediating by macrophages [28], and gonad subdividing by vascularization [27] all play critical roles in testis cord formation and fetal testis morphogenesis. Normal vascular morphogenesis was detected in both the mutant and control testes, and the vascular smooth muscle cells (VSMCs) were differentiated and proliferated normally in *Wt1*^SC-cKO^ mutant testes during fetal testis development, illustrating the disorganization of testis cords found in *Wt1*^SC-cKO^ testes was not the result of vasculature failure. However, the Notch signaling was down-regulated in vasculature-associated interstitial cells in *Wt1*^SC-cKO^ testes. Notch is a transmembrane receptor known to mediate local communication between cells and it is involved in cell fate determination [52]. Loss of Notch function in fetal testes leads to FLC differentiation, whereas gain of the Notch function maintains interstitial progenitor cells and restricts FLC differentiation [23, 24]. Notch signaling balances the FLC differentiation and interstitial progenitor cell maintenance in developing testes. Thus, the decrease of Notch signaling possibly leads to a shift of modulating interstitial progenitor cells to alter FLC fate in *Wt1*^SC-cKO^ testes. Thus, over-differentiation of FLCs likely over-consume the interstitial progenitor cell pool which is proposed to differentiate to PMC as well. The net result thus reduces the number of PMCs in *Wt1*^SC-cKO^ testes.

In summary, we provide evidence illustrating the role of *Wt1* in regulating testis growth and organization in fetal and newborn mice through the development of PMCs and FLCs. A deletion of *Wt1* in Sertoli cells leads to a considerably reduction in PMC differentiation, concomitant with aberrant FLC development, and these changes impede the relative populations of somatic cells in the testes to confer proper testis compartmentalization in *Wt1*^SC-cKO^ testes. In this study, we have provided compelling evidence illustrating that fetal testis compartmentalization relies on the intricate interactions between three somatic cell types, namely Sertoli cell, PMCs and FLCs, during fetal testis development.

**Declaration of interest:** the authors have nothing to disclose.

### Funding

This study was supported by the Major Research Plan “973” Project (2011CB944302 and 2012CB944702), the National Technology Support Project (2012DAI131B08), and the National Nature Science Foundation of China (31471352, 31471400 and 31171380).

## Supporting Information

S1 Fig Original images of Fig 1A (control). Immunofluorescence analysis of peritubular myoid cell (PMC) marker α-SMA (FITC, green fluorescence) and proliferation marker PCNA (TRITC, red fluorescence) in cross-sections of control mouse testes in E13.5 to E18.5.
(PDF)

S2 Fig Original images of Fig 1A (*Wt1*^SC-cKO^). Immunofluorescence analysis of α-SMA (FITC, green fluorescence) and PCNA (TRITC, red fluorescence) in cross-sections of *Wt1*^SC-cKO^ mouse testes in E13.5 to E18.5.
(PDF)

S3 Fig Original images of Fig 1F. Immunofluorescence analysis of α-SMA (TRITC, red fluorescence) and apoptotic analysis (TUNEL assay, green fluorescence) in E14.5 to E18.5 control and *Wt1*^SC-cKO^ testes.
(PDF)

S4 Fig Original images of Fig 2A (control). Immunofluorescence analysis of fetal Leydig cell (FLC) marker HSD3B1 (FITC, green fluorescence) and proliferation marker Ki67 (TRITC, red fluorescence) in cross-sections of control mouse testes in E13.5 to E18.5.
(PDF)

S5 Fig Original images of Fig 2A (*Wt1*^SC-cKO^). Immunofluorescence analysis of HSD3B1 (FITC, green fluorescence) and Ki67 (TRITC, red fluorescence) in cross-sections of *Wt1*^SC-cKO^ mouse testes in E13.5 to E18.5.
(PDF)

S6 Fig Original images of Fig 2F. Immunofluorescence analysis of HSD3B1 (TRITC, red fluorescence) and apoptotic analysis (TUNEL assay, green fluorescence) in E14.5 to E18.5 control and *Wt1*^SC-cKO^ testes.
(PDF)

S7 Fig Original images of Fig 3A (control). Immunofluorescence analysis of HSD3B1 (FITC, green fluorescence) and α-SMA (TRITC, red fluorescence) in cross-sections of control mouse testes in E13.5 to E18.5.
(PDF)

S8 Fig Original images of Fig 3A (*Wt1*^SC-cKO^). Immunofluorescence analysis of HSD3B1 (FITC, green fluorescence) and α-SMA (TRITC, red fluorescence) in cross-sections of *Wt1*^SC-cKO^ mouse testes in E13.5 to E18.5.
(PDF)

S9 Fig Original images of Fig 4A. Immunofluorescence analysis of uncharacterized interstitial progenitor cell marker VCAM1 (TRITC, red fluorescence) and FLC marker CYP11A1 (FITC, green fluorescence) in cross-sections of control *vs*. *Wt1*^SC-cKO^ mouse testes in E14.5, E16.5 and E18.5.
(PDF)

S10 Fig Original images of Fig 5A. Immunofluorescence analysis of vascular smooth muscle cell (VSMC) marker α-SMA (FITC, green fluorescence) and proliferation marker PCNA (TRITC, red fluorescence) in cross-sections of control *vs*. *Wt1*^SC-cKO^ mouse testes in E14.5, E16.5 and E18.5.
(PDF)

S11 Fig Original images of Fig 5C. Immunofluorescence analysis of VSMC marker α-SMA (FITC, green fluorescence) and apoptotic analysis (TUNEL assay, green fluorescence) in cross-sections of control *vs*. *Wt1*^SC-cKO^ mouse testes in E14.5, E16.5 and E18.5.
(PDF)

S1 Table Primer pairs used for qPCR to assess the steady-state mRNA level of target genes. (DOC)
