# Supplementary figures and images for: Sertoli Cell Wt1 Regulates Peritubular Myoid Cell and Fetal Leydig Cell Differentiation during Fetal Testis Development
Source: PLoS One. 2016 Dec 30;11(12):e0167920. doi: 10.1371/journal.pone.0167920 (PMC5201236; doi:10.1371/journal.pone.0167920)

S1\_Fig (Wen Q et al.)

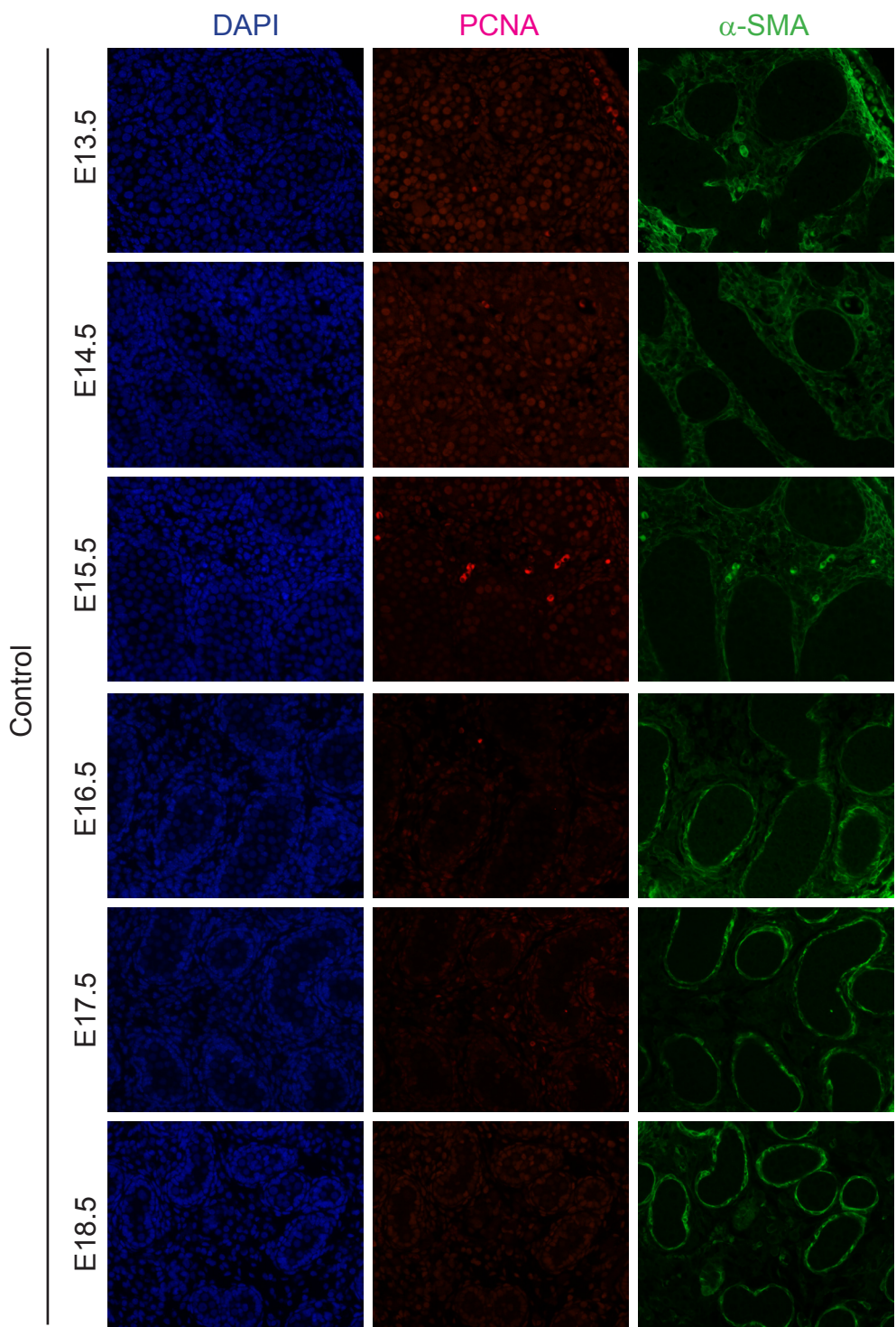

Supplement: S1 Fig — Immunofluorescence analysis of peritubular myoid cell (PMC) marker α-SMA (FITC, green fluorescence) and proliferation marker PCNA (TRITC, red fluorescence) in cross-sections of control mouse testes in E13.5 to E18.5. (PDF) [file pone.0167920.s001.pdf]

S2\_Fig (Wen Q et al.)

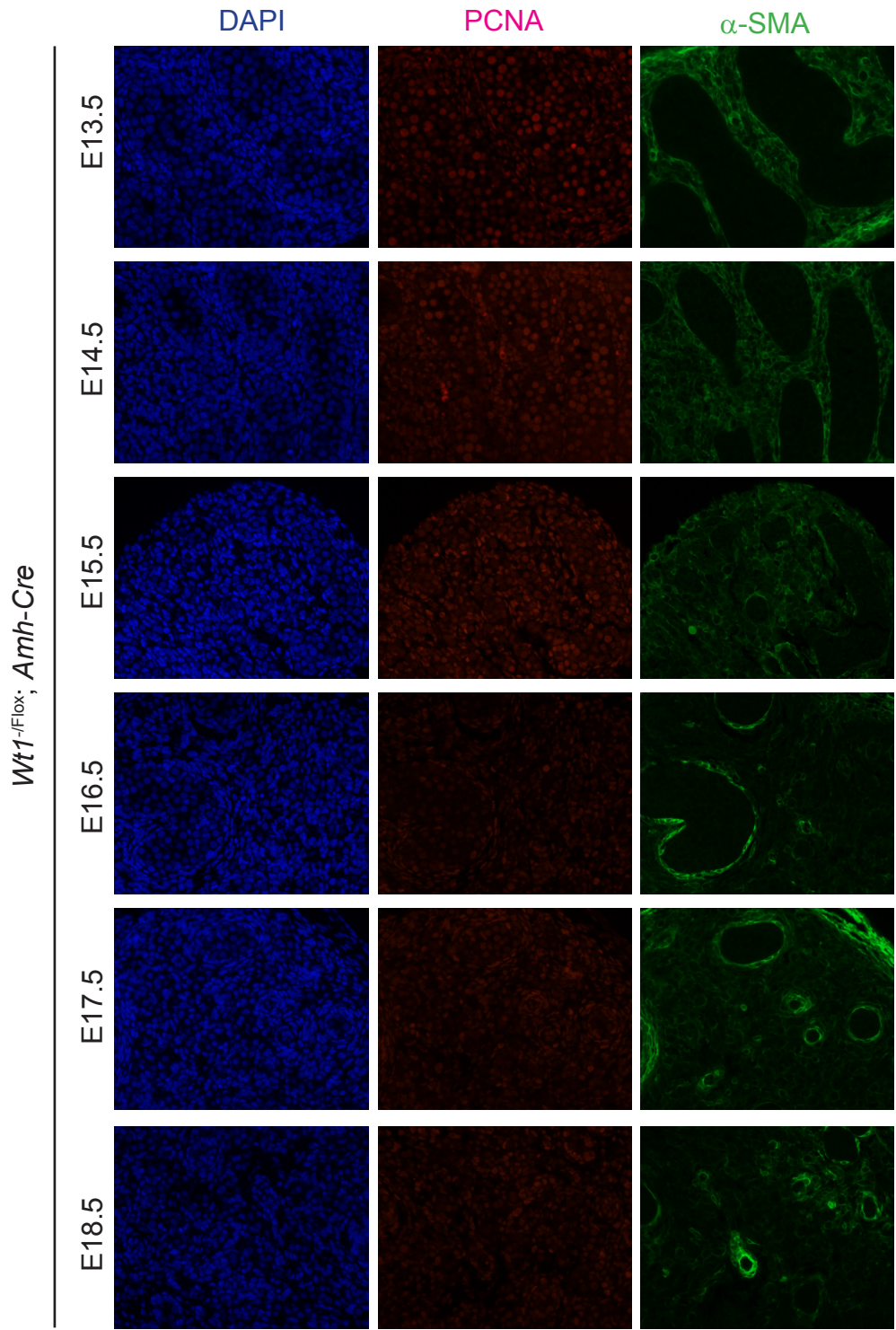

Supplement: S2 Fig — Immunofluorescence analysis of α-SMA (FITC, green fluorescence) and PCNA (TRITC, red fluorescence) in cross-sections of Wt1SC-cKO mouse testes in E13.5 to E18.5. (PDF) [file pone.0167920.s002.pdf]

S3\_Fig (Wen Q et al.)

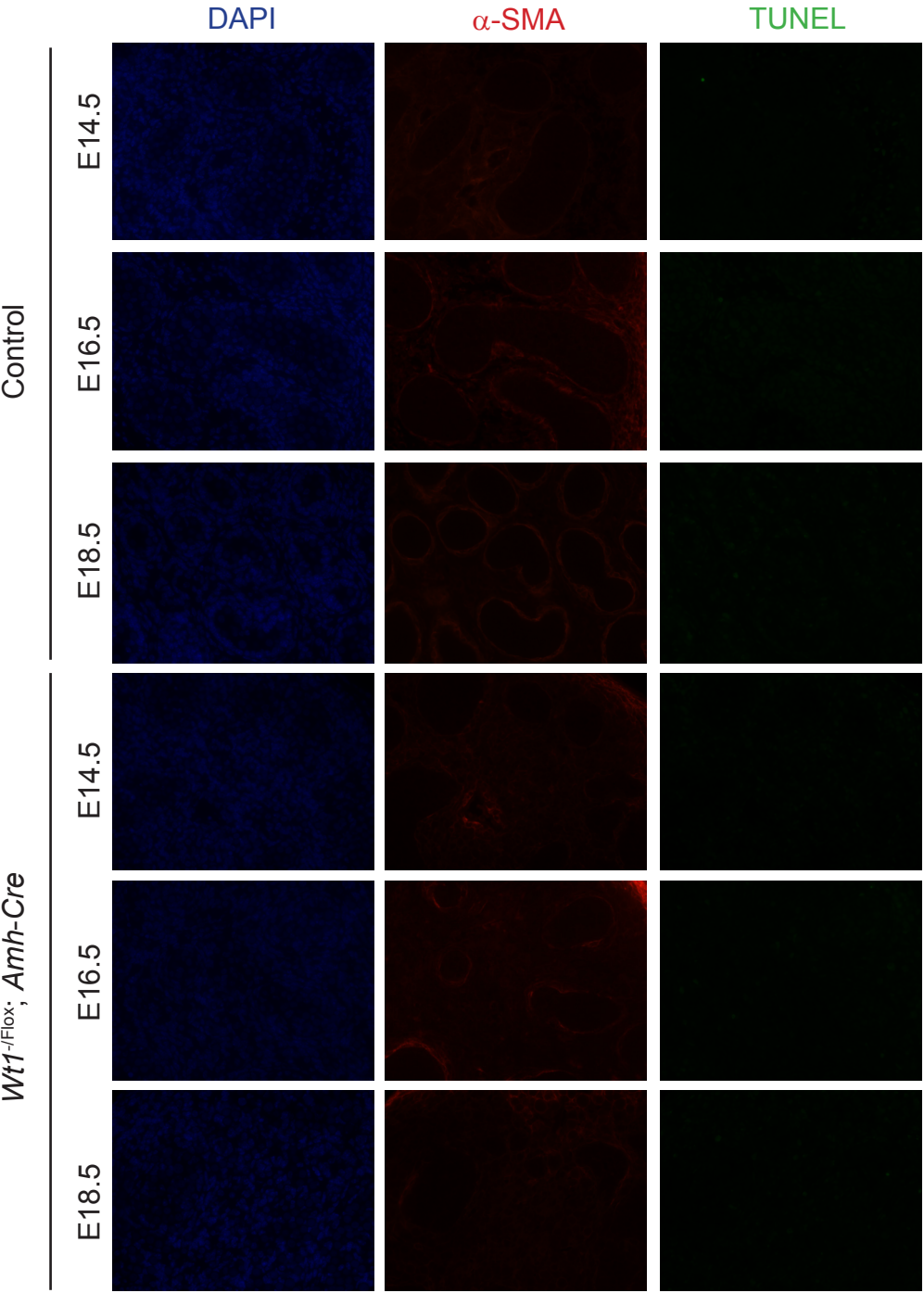

Supplement: S3 Fig — Immunofluorescence analysis of α-SMA (TRITC, red fluorescence) and apoptotic analysis (TUNEL assay, green fluorescence) in E14.5 to E18.5 control and Wt1SC-cKO testes. (PDF) [file pone.0167920.s003.pdf]

S4\_Fig (Wen Q et al.)

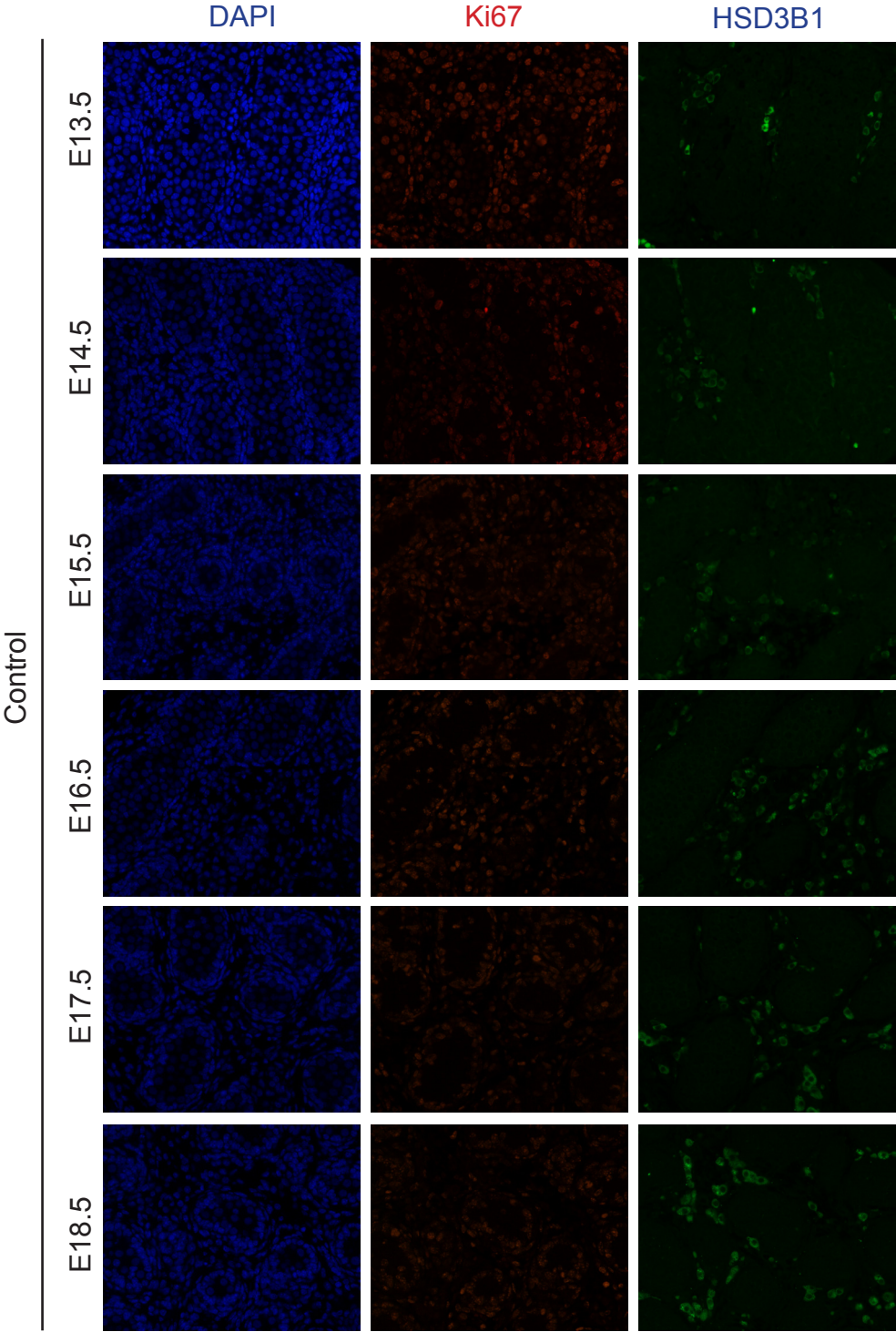

Supplement: S4 Fig — Immunofluorescence analysis of fetal Leydig cell (FLC) marker HSD3B1 (FITC, green fluorescence) and proliferation marker Ki67 (TRITC, red fluorescence) in cross-sections of control mouse testes in E13.5 to E18.5. (PDF) [file pone.0167920.s004.pdf]

S5\_Fig (Wen Q et al.)

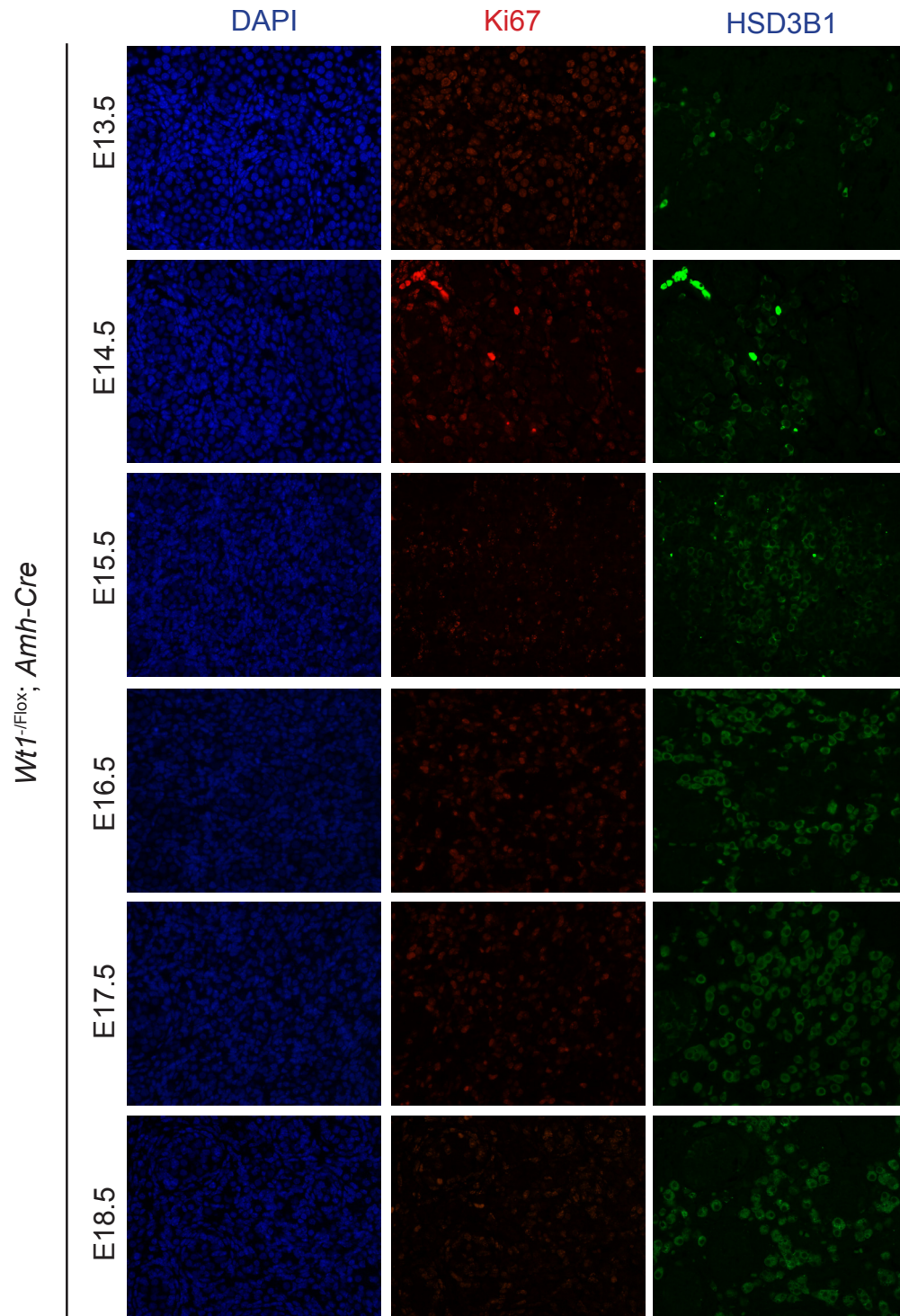

Supplement: S5 Fig — Immunofluorescence analysis of HSD3B1 (FITC, green fluorescence) and Ki67 (TRITC, red fluorescence) in cross-sections of Wt1SC-cKO mouse testes in E13.5 to E18.5. (PDF) [file pone.0167920.s005.pdf]

S6\_Fig (Wen Q et al.)

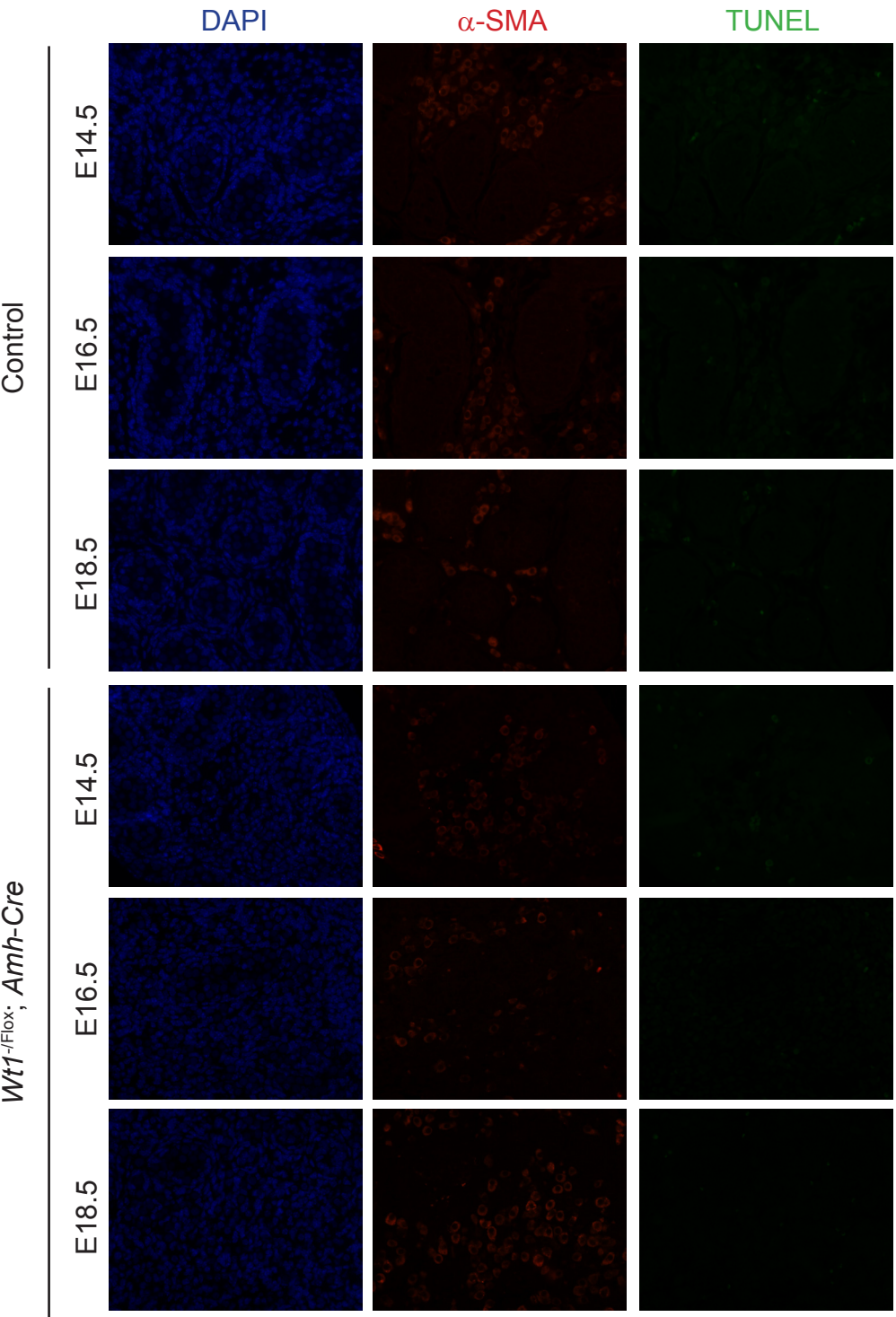

Supplement: S6 Fig — Immunofluorescence analysis of HSD3B1 (TRITC, red fluorescence) and apoptotic analysis (TUNEL assay, green fluorescence) in E14.5 to E18.5 control and Wt1SC-cKO testes. (PDF) [file pone.0167920.s006.pdf]

S7\_Fig (Wen Q et al.)

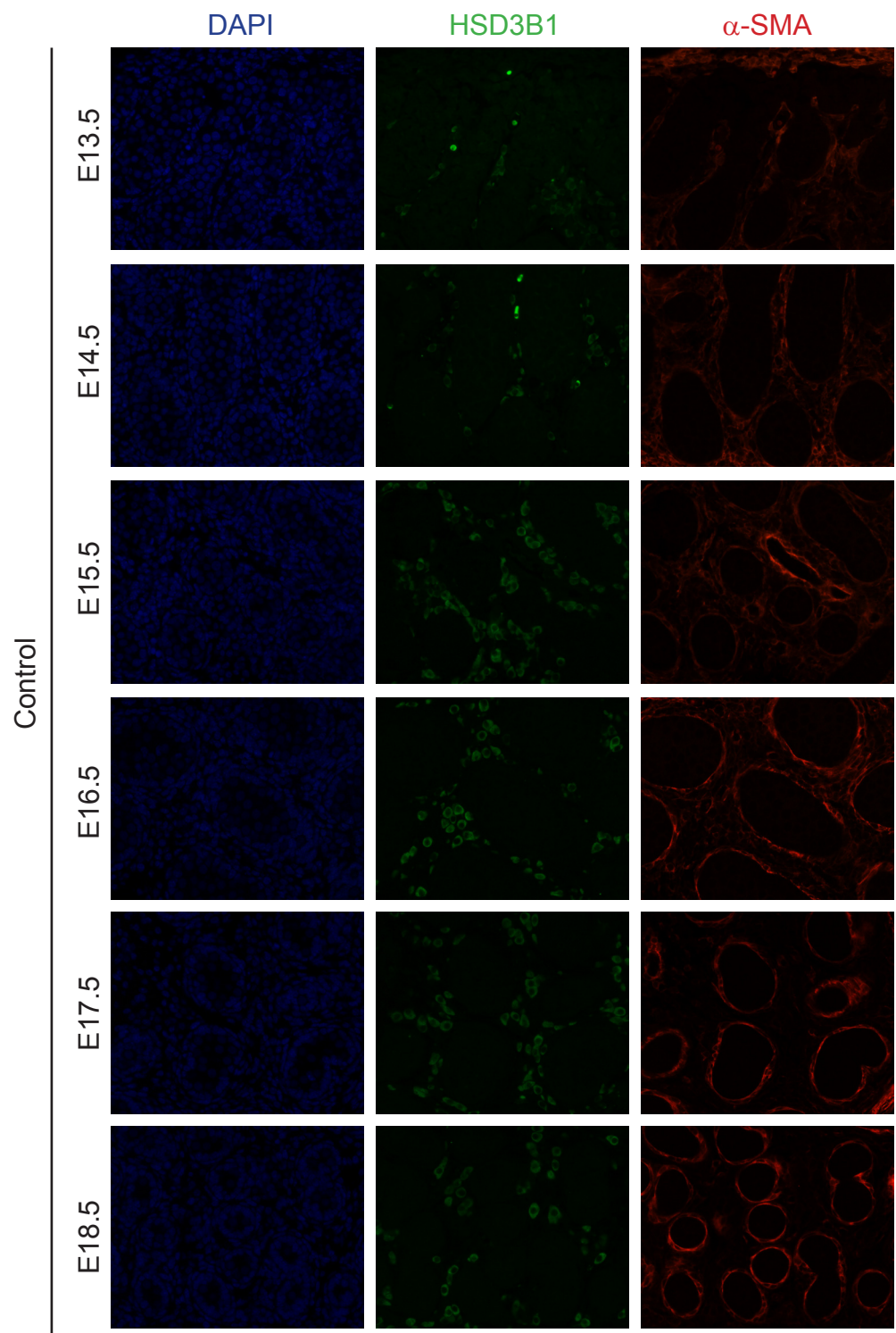

Supplement: S7 Fig — Immunofluorescence analysis of HSD3B1 (FITC, green fluorescence) and α-SMA (TRITC, red fluorescence) in cross-sections of control mouse testes in E13.5 to E18.5. (PDF) [file pone.0167920.s007.pdf]

S8\_Fig (Wen Q et al.)

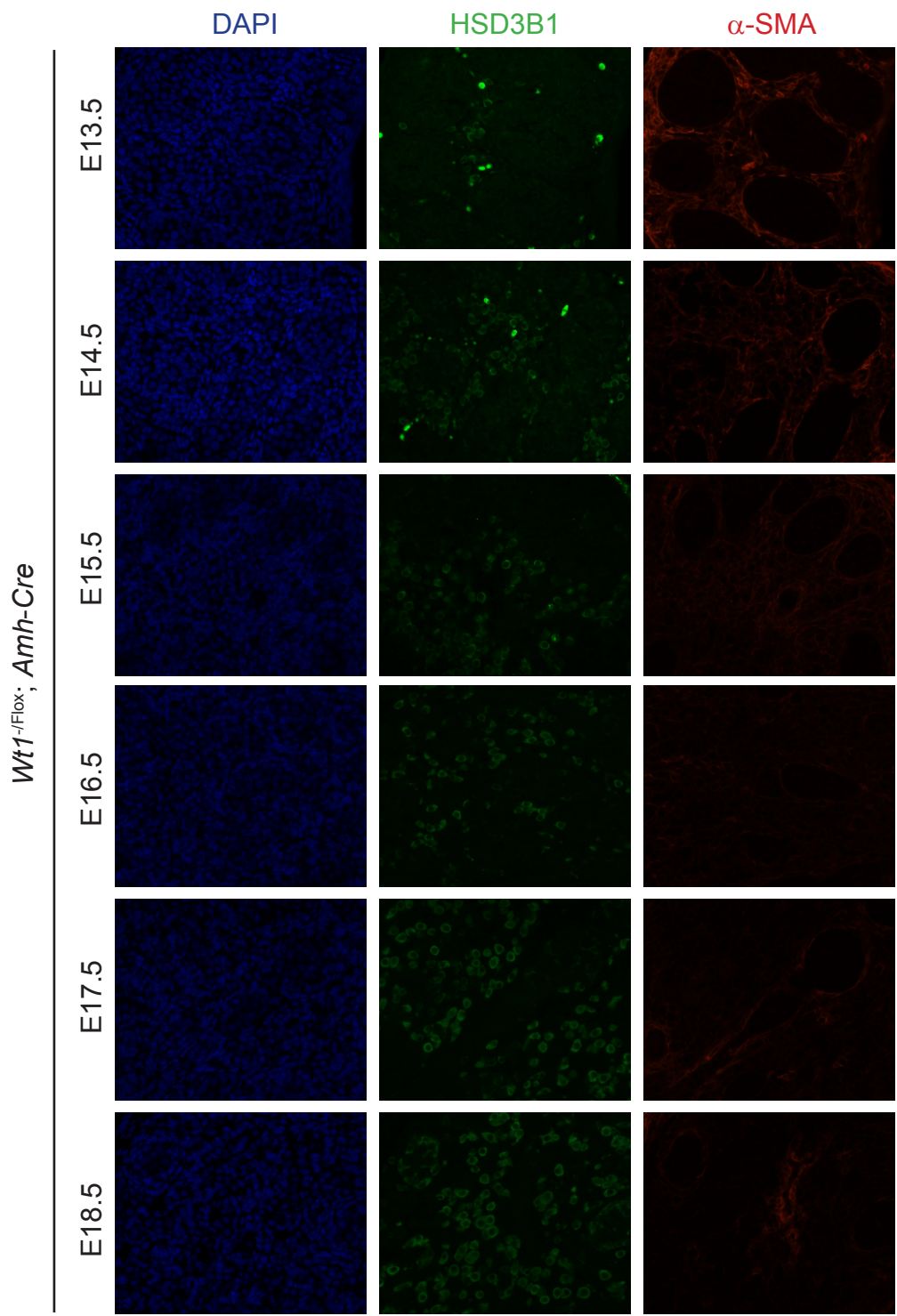

Supplement: S8 Fig — Immunofluorescence analysis of HSD3B1 (FITC, green fluorescence) and α-SMA (TRITC, red fluorescence) in cross-sections of Wt1SC-cKO mouse testes in E13.5 to E18.5. (PDF) [file pone.0167920.s008.pdf]

S9\_Fig (Wen Q et al.)

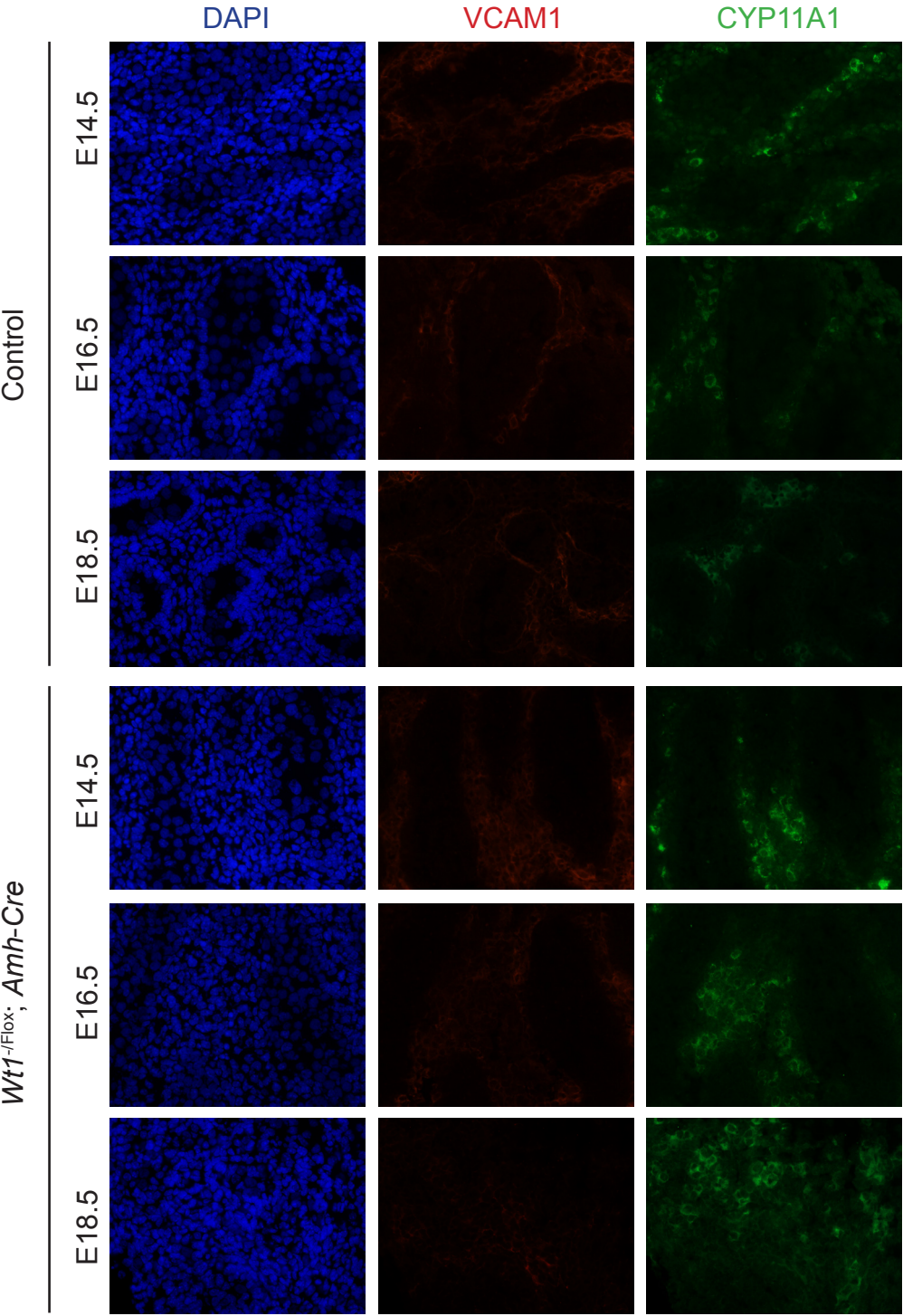

Supplement: S9 Fig — Immunofluorescence analysis of uncharacterized interstitial progenitor cell marker VCAM1 (TRITC, red fluorescence) and FLC marker CYP11A1 (FITC, green fluorescence) in cross-sections of control vs. Wt1SC-cKO mouse testes in E14.5, E16.5 and E18.5. (PDF) [file pone.0167920.s009.pdf]

S10\_Fig (Wen Q et al.)

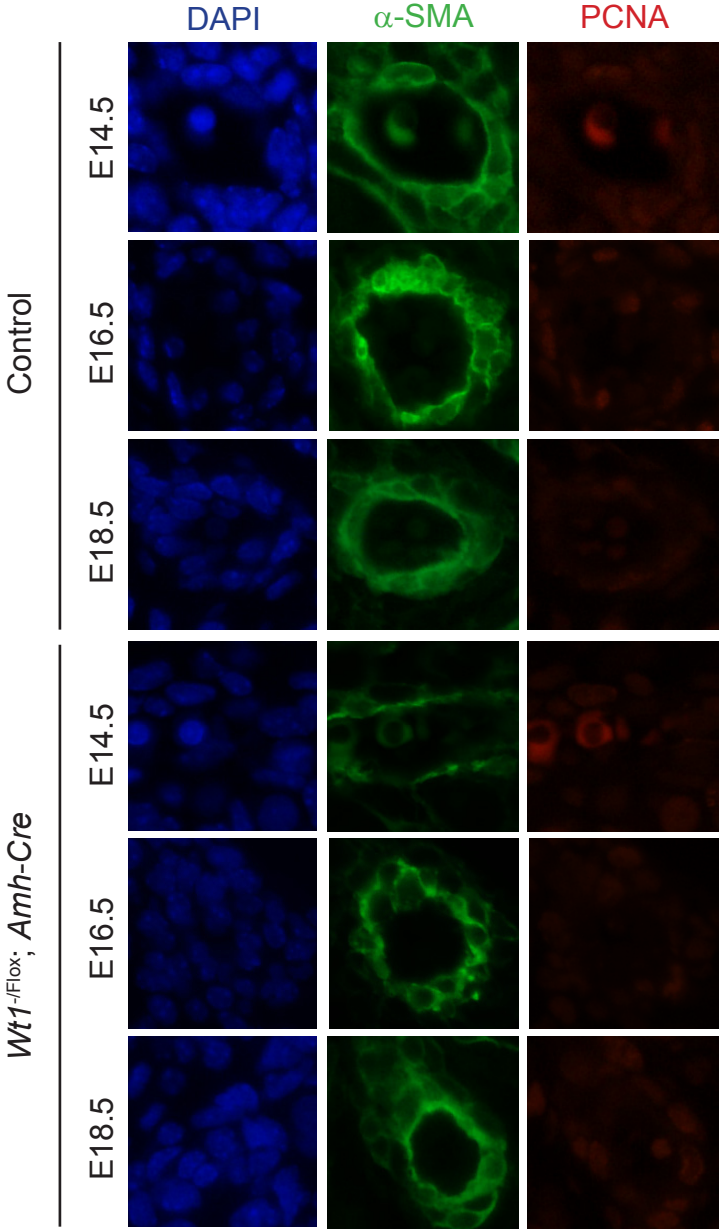

Supplement: S10 Fig — Immunofluorescence analysis of vascular smooth muscle cell (VSMC) marker α-SMA (FITC, green fluorescence) and proliferation marker PCNA (TRITC, red fluorescence) in cross-sections of control vs. Wt1SC-cKO mouse testes in E14.5, E16.5 and E18.5. (PDF) [file pone.0167920.s010.pdf]

S11\_Fig (Wen Q et al.)

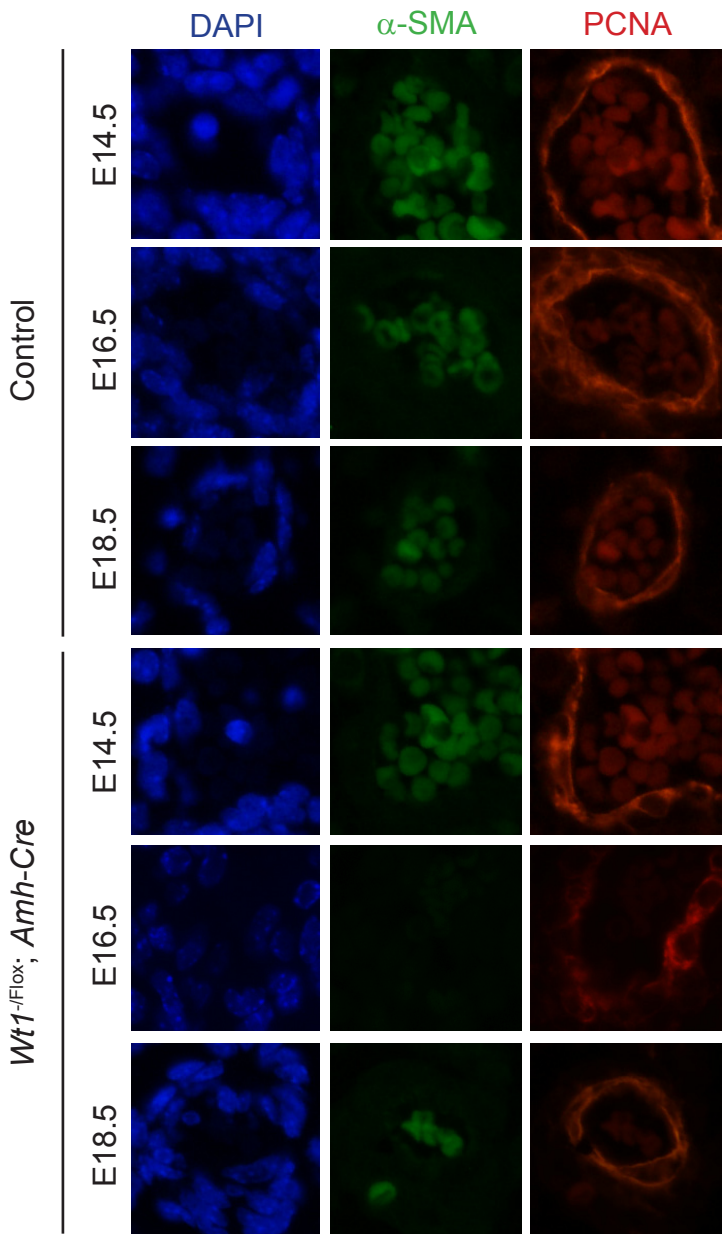

Supplement: S11 Fig — Immunofluorescence analysis of VSMC marker α-SMA (FITC, green fluorescence) and apoptotic analysis (TUNEL assay, green fluorescence) in cross-sections of control vs. Wt1SC-cKO mouse testes in E14.5, E16.5 and E18.5. (PDF) [file pone.0167920.s011.pdf]
