# Supplementary material for: Sertoli Cell Wt1 Regulates Peritubular Myoid Cell and Fetal Leydig Cell Differentiation during Fetal Testis Development
Source: PLoS One. 2016 Dec 30;11(12):e0167920. doi: 10.1371/journal.pone.0167920 (PMC5201236; doi:10.1371/journal.pone.0167920)
Supplement: S1 Table — (DOC) [file pone.0167920.s012.doc]

**S1 Table. Primer pairs used for qPCR to assess the steady-state mRNA level of target genes**

| Gene | GenBank Accession # | Primer pairs (5’-3’) | Amplified Product (bp) |
| --- | --- | --- | --- |
| *Star* | NM_011485.4 | Forward, CCGGAGCAGAGTGGTGTCA  Reverse, CAGTGGATGAAGCACCATGC | 62 |
| *Cyp11a1* | NM_019779.3 | Forward, CCAGTGTCCCCATGCTCAAC  Reverse, TGCATGGTCCTTCCAGGTCT | 73 |
| *Cyp17a1* | NM_007809.3 | Forward, CTTGTCGGACCAAGGAAAAGGCGT  Reverse, CAACCACGGGAATATGTCCACCAG | 348 |
| *Hsd3b1* | NM_008293.3 | Forward, AATCTGAAAGGTACCCAGAA  Reverse, TCATCATAGCTTTGGTGAGG | 499 |
| *α-SMA* | NM_007392.3 | Forward, GAGAAGCCCAGCCAGTCG  Reverse, CTCTTGCTCTGGGCTTCA | 239 |
| *Myh11* | NM_001161775.1 | Forward, CTGCACAACCTGAGGGAGCGATACT  Reverse, AATGGCATAGATGTGAGGCGGC | 167 |
| *Des* | NM_010043.2 | Forward, GACTCCCTGATGAGGCAGATGAGG  Reverse, CCTCGCTGACAACCTCTCCATCCC | 357 |
| *Arx* | NM_007492.3 | Forward, CAAGGATGGTGAGGACAGC  Reverse, TCTGGAACCACACCTGGACT | 202 |
| *Lhx9* | NM_001025565.2 | Forward, TGGGAGTGGACATCGTGAATT  Reverse, GAAAGAAGTTCGCATCCGTTTG | 118 |
| *Vcam1* | NM_011693.3 | Forward, TGCCGAGCTAAATTACACATTG  Reverse, CCTTGTGGAGGGATGTACAGA | 122 |
| *Ptch1* | NM_008957.2 | Forward, AAAGAACTGCGGCAAGTTTTTG  Reverse, CTTCTCCTATCTTCTGACGGGT | 163 |
| *Pdgfrα* | NM_001083316.1 | Forward, TCCATGCTAGACTCAGAAGTCA  Reverse, TCCCGGTGGACACAATTTTTC | 139 |
| *Nes* | NM_016701.3 | Forward, GCTGGAACAGAGATTGGAAGG  Reverse, CCAGGATCTGAGCGATCTGAC | 124 |
| *Cdh5* | NM_009868.4 | Forward, TCCTCTGCATCCTCACTATCACA  Reverse, GTAAGTGACCAACTGCTCGTGAAT | 121 |
| *Jag1* | NM_013822.5 | Forward, TGACATGGATAAACACCAGCA  Reverse, GCAGCCCACTGTCTGCTATAC | 195 |
| *Notch2* | NM_010928.2 | Forward, ATGCACCATGACATCGTTCG  Reverse, GATAGAGTCACTGAGCTCTCG | 286 |
| *Notch3* | NM_008716.2 | Forward, GCTTGGGAAATCTGCCTTAC  Reverse, GAGCAATGGCCCTAAGCCAT | 318 |
| *Hes1* | NM_008235.2 | Forward, ACGTGCGAGGGCGTTAATAC  Reverse, ATTGATCTGGGTCATGCAGTTG | 73 |
| *Gapdh* | NM_001289726.1 | Forward, TTGTCTCCTGCGACTTCAACA  Reverse, ACCAGGAAATGAGCTTGACAAAG | 98 |
